# Chemical vapour deposition (CVD) of nickel oxide using the novel nickel dialkylaminoalkoxide precursor [Ni(dmamp′)_2_] (dmamp′ = 2-dimethylamino-2-methyl-1-propanolate)†

**DOI:** 10.1039/d1ra03263a

**Published:** 2021-06-23

**Authors:** Rachel L. Wilson, Thomas J. Macdonald, Chieh-Ting Lin, Shengda Xu, Alaric Taylor, Caroline E. Knapp, Stefan Guldin, Martyn A. McLachlan, Claire J. Carmalt, Chris S. Blackman

**Affiliations:** Department of Chemistry, University College London 20 Gordon Street London WC1H 0AJ UK c.blackman@ucl.ac.uk; Department of Chemistry, Center for Plastic Electronics, Imperial College London 80 Wood Lane London W12 0BZ UK; Department of Materials, Center for Plastic Electronics, Imperial College London Exhibition Road London SW7 2AZ UK; Department of Chemical Engineering, University College London Torrington Place London WC1E 7JE UK; London Centre for Nanotechnology 17-19 Gordon Street WC1H 0AH UK

## Abstract

Nickel oxide (NiO) has good optical transparency and wide band-gap, and due to the particular alignment of valence and conduction band energies with typical current collector materials has been used in solar cells as an efficient hole transport-electron blocking layer, where it is most commonly deposited *via* sol–gel or directly deposited as nanoparticles. An attractive alternative approach is *via* vapour deposition. This paper describes the chemical vapour deposition of p-type nickel oxide (NiO) thin films using the new nickel CVD precursor [Ni(dmamp′)_2_], which unlike previous examples in literature is synthesised using the readily commercially available dialkylaminoalkoxide ligand dmamp′ (2-dimethylamino-2-methyl-1-propanolate). The use of vapour deposited NiO as a blocking layer in a solar-cell device is presented, including benchmarking of performance and potential routes to improving performance to viable levels.

## Abstract 2

We describe CVD of nickel oxide (NiO) thin films using a new precursor [Ni(dmamp′)_2_], synthesised using a readily commercially available dialkylaminoalkoxide ligand (dmamp′), which is applied to synthesis of a hole transport-electron blocking layer.

## Graphical abstract

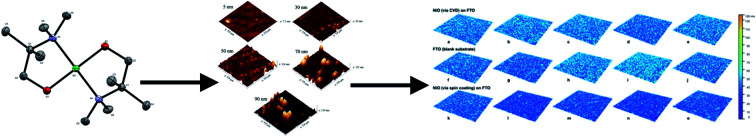

## Introduction

Among p-type semiconductors, nickel oxide (NiO) has good optical transparency and wide band-gap, which result in low visible light absorption losses, and due to the particular alignment of valence and conduction band energies with typical current collector materials it has been widely used in solar cells as an efficient hole transport-electron blocking layer.^1^ NiO in solar cells is most commonly deposited *via* sol–gel and annealed^2^ or directly deposited as nanoparticles without annealing,^3^ although an attractive alternative approach is *via* vapour deposition.

Vapour deposited thin films of the p-type semiconductor nickel oxide (NiO) have previously been prepared using a range of techniques including metal–organic chemical vapour deposition (MO-CVD),^4–6^ nebulised spray pyrolysis (NSP),^7^ electron-beam evaporation and atomic layer deposition (ALD).^8–11^ Our extensive attempts at producing NiO using a simple ALD process with water as the oxidant, making use of various precursors reported in literature, were uniformly unsuccessful. We were therefore interested in using a CVD route to thin films of NiO, in which the use of nickel(ii) aminoalkoxides as precursors are most commonly reported. Nickel(ii) aminoalkoxides such as [Ni(dmamp)_2_] (dmamp = 2-dimethylamino-1-methyl-1-propanolate), [Ni(deamp)_2_] (deamp = 1-diethylamino-2-methyl-2-propanolate), [Ni(emamp)_2_] (emamp = 1-ethylmethylamino-2-methyl-2-propanolate) and [Ni(dmamb)_2_] (dmamb = 1-dimethylamino-2-methyl-2-butanolate), have been employed as precursors for metal–organic chemical vapour deposition (MOCVD) of nickel metal thin films,^4^ and atomic layer deposition (ALD) of NiO thin films.^8^ The volatile liquid precursor nickel bis(1-dimethylamino-2-methyl-2-butanolate), [Ni(dmamb)_2_] has been employed as an MOCVD precursor^5^ and an ALD precursor with water^8^ for the deposition of NiO at temperatures in the range of 230–410 °C and 140–240 °C, respectively. There are several publications outlining the use of similar [Ni(dmamp)_2_]-type structures for use in MOCVD^5^ and ALD^12^ applications to deposit NiO thin films. Although solids at room temperature, nickel dialkylaminoalkoxide complexes ([Ni(dmamp)_2_], [Ni(emamp)_2_], and [Ni(deamp)_2_]) are volatile and thermally stable. However none of the aforementioned dmamp, deamp, emamp or dmamb free ligands were freely commercially available in order to synthesise these previously demonstrated CVD precursors.

Consequently this paper describes the chemical vapour deposition of p-type nickel oxide (NiO) thin films using the new nickel CVD precursor [Ni(dmamp′)_2_], which unlike previous examples is synthesised using the readily available dialkylaminoalkoxide ligand dmamp′ (2-dimethylamino-2-methyl-1-propanolate), providing a CVD precursor to NiO that is more easily accessible for other researchers than existing examples in literature. We also describe the use of the deposited NiO thin films as a blocking layer in a solar-cell device, including benchmarking of performance.

## Results and discussion

### Precursor synthesis

**Fig. 1:**
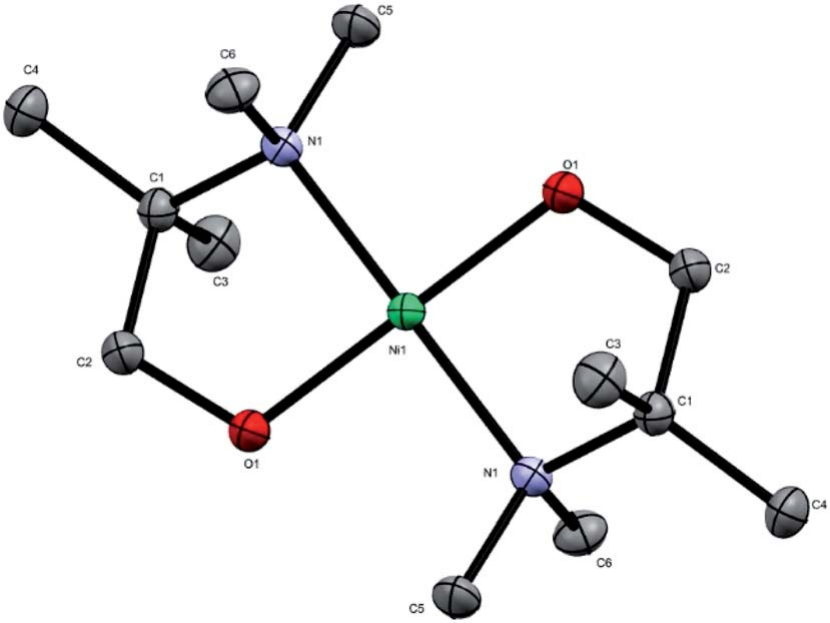
Crystal structure of [Ni(dmamp′)_2_]. Atoms shown as thermal ellipsoids; carbon in grey, nitrogen in blue, oxygen in red and nickel in green. Hydrogen atoms omitted for clarity. CCDC deposition number 1978578.†

The novel precursor [Ni(dmamp′)_2_] was synthesised following adaptations from two different literature reports in relatively high yields (∼86%);^4,13^ recrystallisation in THF produced orange crystals of the orthorhombic space group with square planar geometry at the nickel centre, as confirmed by single crystal X-ray crystallography. The molecular structure of [Ni(dmamp′)_2_] (Fig. 1) shows a nickel centre bound to 2 oxygen atoms and 2 nitrogen atoms. The O(1)–Ni(1)–O(1)^1^ and N(1)–Ni(1)–N(1)^1^ bond angles were both 180° which confirms the square planar geometry of the nickel centre. Both of the Ni–O and Ni–N bond lengths were 1.84 Å and 1.95 Å, respectively which are comparable with data reported in the literature.^14^ Bond angles for similar tetra-coordinated nickel complexes have been reported. Hubert-Pfalzgraf *et al.* reported bond lengths of 1.93 Å and 2.10 Å for Ni–O and Ni–N respectively,^13^ and Yoo *et al.* reported values of 1.83 Å and 1.93 Å for Ni–O and Ni–N respectively.^4^ Similarly, the C–N and C–C bond lengths within the dmamp′ ligands are also comparable to those outlined in these papers. The structure was consistent with the ^1^H and ^13^C NMR spectra.

**Fig. 2:**
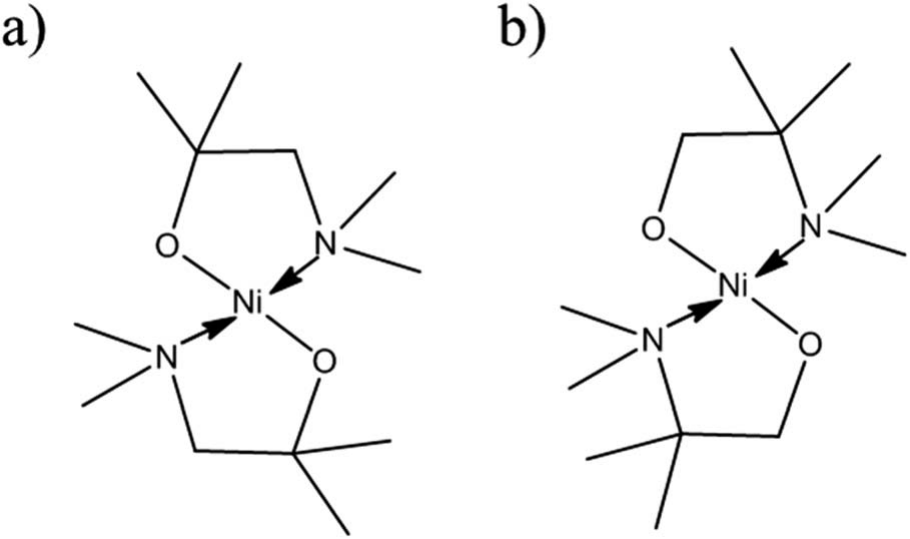
Structure of [Ni(dmamp)_2_] synthesised in (a) literature^13^ and (b) this work.

The [Ni(dmamp′)_2_] synthesised in this work is different to similar precursors reported in the literature^13^ because the two methyl groups on the dmamp′ ligand backbone are positioned on a different carbon atom (Fig. 2).

Thermal analysis of the novel precursor [Ni(dmamp′)_2_] suggests a melting point of 180 °C (differential scanning calorimetry (DSC) curve, blue line) although thermogravimetric analysis (TGA) indicates mass loss is observed from significantly below that temperature. Subsequently, decomposition occurs in the range of ∼200–250 °C, with an overall mass loss of ∼75% (Fig. S1†). The data correlate well with that reported in the literature for similar dmamp-type structures.^15^ The temperature range of weight loss for the novel [Ni(dmamp′)_2_] complex is similar to that for [Ni(acac)_2_].^4^

### Thin film deposition

There are several publications outlining the use of similar [Ni(dmamp)_2_]-type structures for use in ALD with both H_2_O^15^ and O_2_ (ref. 16) as co-reactants to deposit NiO thin films. Although solids at room temperature, these nickel dialkylamino alkoxide complexes ([Ni(dmamp)_2_], [Ni(emamp)_2_], and [Ni(deamp)_2_]) are volatile and thermally stable. Although the temperature range of decomposition for this novel precursor is comparable to those reported in the literature for similar [Ni(dmamp)_2_]-type structures, it is believed that the novel precursor has a higher thermal stability as its end decomposition temperature is greater (240 °C compared to 174 °C in literature),^15^ which suggested it may have preferable properties for ALD deposition. Extensive attempts at producing NiO using a true ALD process, either using precursors previously described in literature for ALD or the novel [Ni(dmamp′)_2_] precursor synthesised here, were however uniformly unsuccessful and therefore we carried out CVD in order to target thin film growth of NiO.

**Fig. 3:**
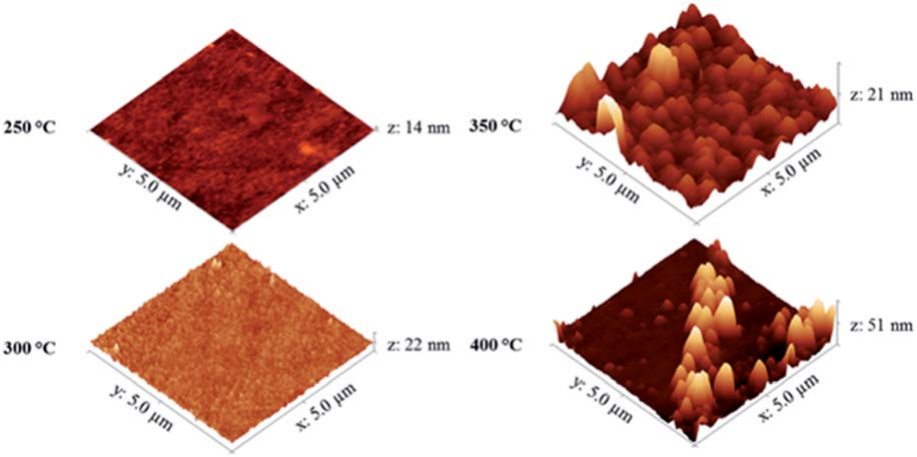
AFM images of NiO films deposited by CVD of [Ni(dmamp′)_2_] at different substrate temperatures.

TGA/DSC showed that precursor vaporisation occurred from as little as 80 °C, and initial experiments indicated at this temperature the vapour pressure was sufficient for vapour transport of the precursor into the reactor without causing precursor decomposition. ^1^H NMR analysis of the precursor after being heated at 80 °C for long periods of time (several weeks) showed no signs of decomposition. In order to determine the optimum growth temperature for the NiO films, CVD experiments were initially performed for 24 hours at growth temperatures of 250–400 °C (the long growth times were due to the relatively low vapour pressure of the precursor at 80 °C). For films deposited at 250–350 °C the refractive index values for the deposited films were in line with those reported in the literature for NiO, which range from 2.3 to 2.9 depending on the deposition temperature,^17^ and increased with film thickness (see ESI†). However for films deposited at 400 °C the value of *n* decreased. Similar behaviour has been reported by Lu *et al.*, where a decrease of 0.2 in the refractive index of NiO films was observed in the photon energy region from 1.2 to 3.3 eV when samples were annealed at temperatures above 500 °C.^17^ The AFM images in Fig. 3 show that as the growth temperature increased, the surface roughness of the NiO films also increases. Some larger particulates were observed on the film surface as the deposition temperature exceeded 300 °C.

**Fig. 4:**
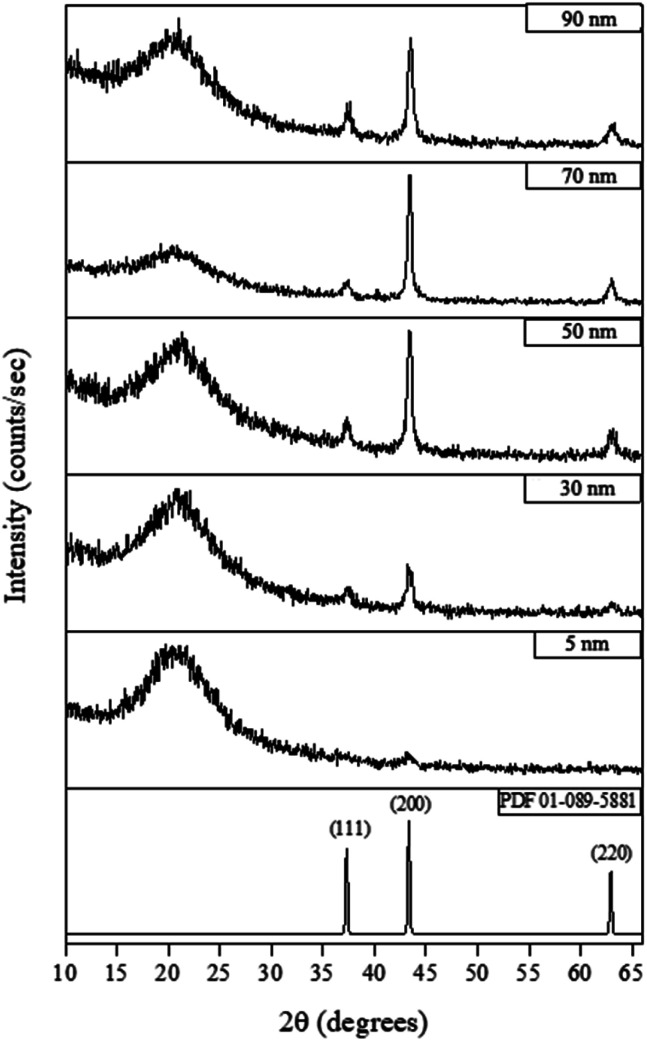
Typical XRD patterns for different thickness of NiO films deposited by CVD from [Ni(dmamp′)_2_] at 300 °C. NiO reference pattern included [PDF 01-089-5881].

Fig. 4 shows the X-ray diffraction (XRD) measurements for films deposited at 300 °C for various lengths of time (see ESI† for details). The XRD patterns show that a 6 hours-deposited CVD film (∼5 nm) is weakly diffracting, most likely because the films were too thin to produce significant diffraction, which is consistent with the appearance of a broad background peak attributed to breakthrough to the glass substrate around 20° 2*θ*. For films deposited for 18 hours or longer (>30 nm), NiO peaks became visible, with the (200) reflection the most intense (NiO PDF reference number 01-089-5881) indicating some degree of preferred orientation. As the film thickness increased (*i.e.* longer deposition time) the intensity of the NiO peaks increased.

**Fig. 5:**
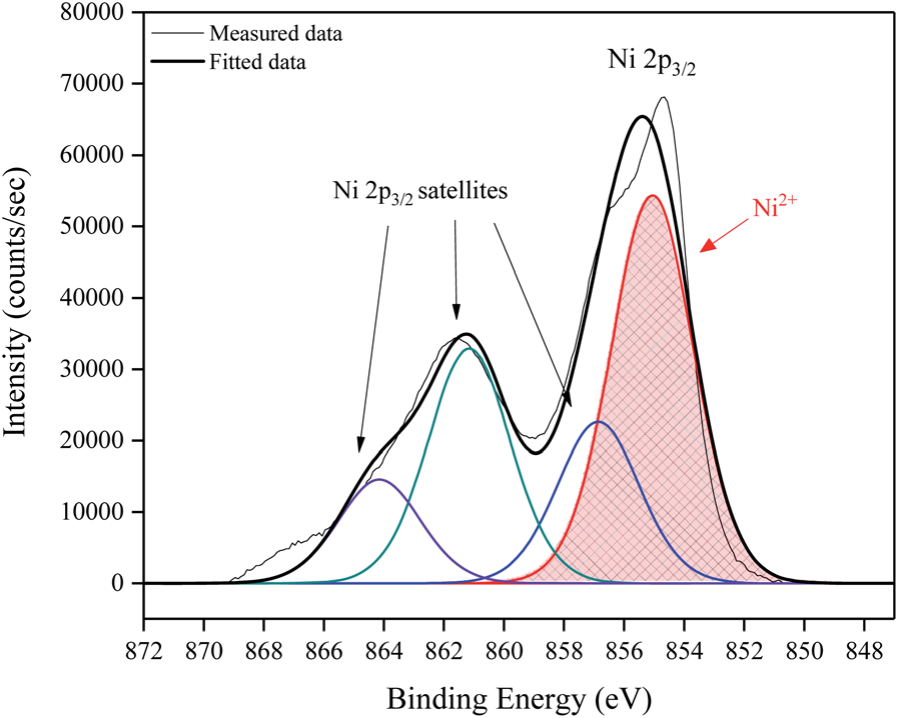
High resolution surface scan of Ni 2p_3/2_ peak with 3 observable satellite intensities fit by broad peaks (FWHM 3.2 eV) with binding energies at 1.8, 6.1 and 9.0 eV above the principal peak at 855.4 eV.

To determine the elemental composition and electronic state of the elements within the NiO films, X-ray photoelectron spectroscopy (XPS) was performed. Fig. 5 shows a high resolution surface scan of the Ni 2p_3/2_ peak. The principal core peak of Ni^2+^ (red peak) was observed at a binding energy of 855.4 ± 0.2 eV, with satellite peaks 6.1 eV (green peak) and 9.0 eV (purple peak) above the principal peak, which can be attributed to multi-electron excitation.^18^ The prominent satellite shoulder 1.8 eV above the Ni 2p_3/2_ principal peak (blue peak) is unique to NiO. It has the same shape and FWHM (3.2 eV) as the principal peak. A typical survey XPS spectrum, full Ni 2p peak envelope and O1s spectrum are provided in ESI.†

**Fig. 6:**
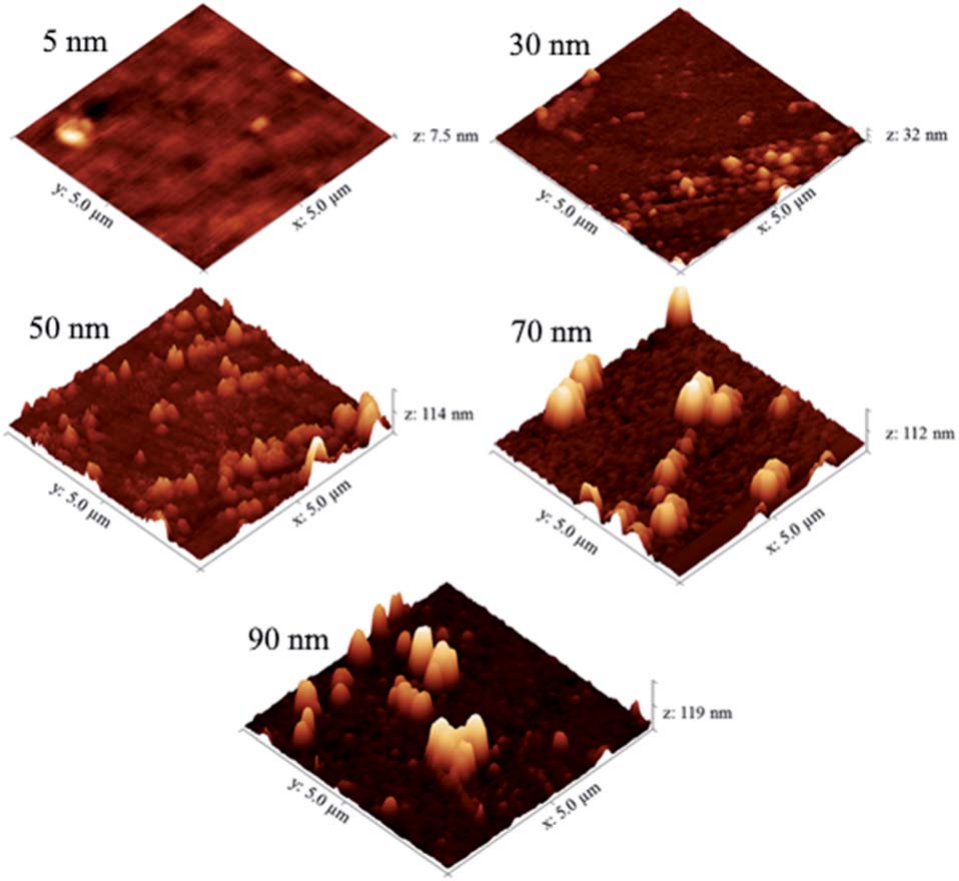
AFM images of different thickness NiO films deposited by CVD of [Ni(dmamp)_2_] at 300 °C. Images set to a physical scale factor of 10 in the *z* axis.

The AFM images in Fig. 6 show that as the deposition time (and hence film thickness) increased, the surface roughness of the NiO films also increases. Some larger particulates were observed on the film surface as the film thickness approached 70 nm. This resulted in a sharp increase in the surface roughness of the films from 10 nm to 20 nm RMS (root mean square) roughness for films of 30 nm and 50 nm thickness respectively.

### Integration in solar cell device

**Fig. 7:**
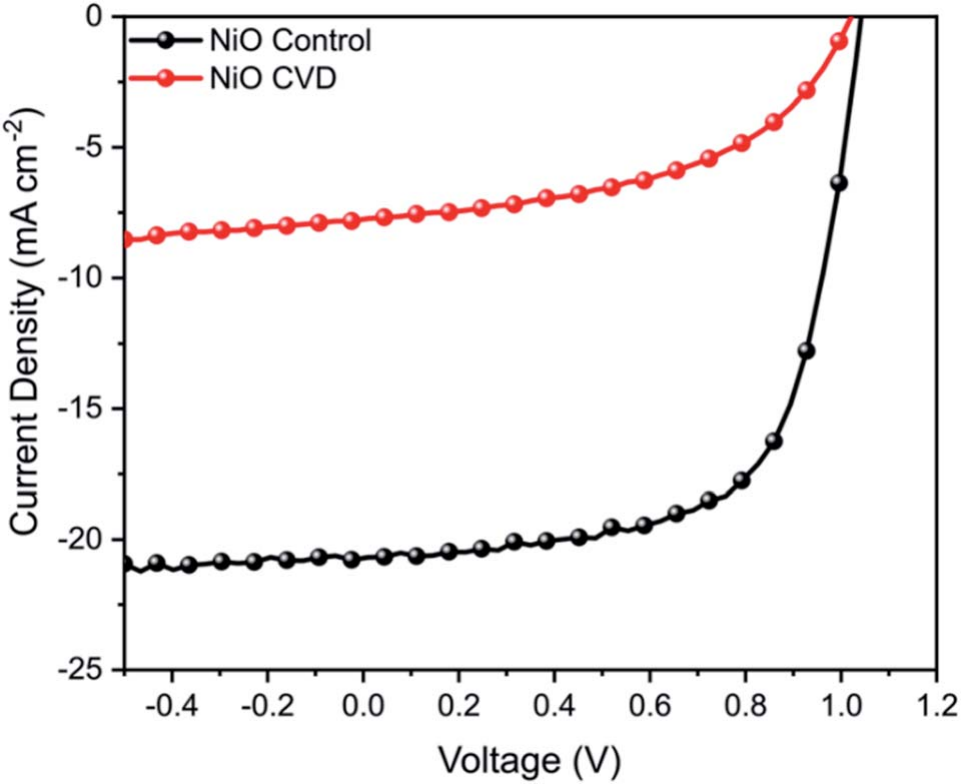
*J*–*V* curve for NiO control and NiO CVD devices measured under AM 1.5 conditions between −0.5 and 1.2 V.

NiO has been used as a bottom hole transport layer (HTL) in inverted perovskite solar cells (p-i-n), as well as a top HTL in conventional perovskite solar cells (n-i-p).^19^ In order to perform functional testing of the CVD prepared thin films, inverted (p-i-n) perovskite solar cells were fabricated. For comparison, NiO control films were prepared by a typical sol–gel method from nickel acetate, analogous to previous reports.^20^ NiO control devices achieved a maximum power conversion efficiency (PCE) of 14.1% (Fig. 7), however the NiO CVD devices had a significantly lower performance (PCE 3.9%).

While NiO is clearly a versatile metal oxide HTL, its surface roughness is of particular importance for p-i-n perovskite solar cells since this has a large influence on the growth of the perovskite crystals (absorber layer) and to achieve smooth, uniform and dense perovskite layers, it is important that the underlying NiO film has low surface roughness (RMS < 5 nm).^21^ In order to determine whether the significant decrease in PV performance could be due to a change in surface roughness from the deposition of NiO on FTO, we performed additional AFM measurements of FTO, CVD-deposited NiO/FTO and spin-coated NiO/FTO (ESI†). A CVD-deposited NiO film on an FTO-coated glass substrate had an RMS roughness of 11.6 ± 0.5 nm, very similar to that of the underlying substrate (11.4 ± 0.2 nm) demonstrating good conformality, however a spin-coated NiO film on an FTO-coated glass substrate had an RMS roughness of 9.7 ± 0.1 nm. While the difference was only a few nanometers, the rougher surface of the CVD-deposited NiO films is thought likely responsible for the drop in performance.^21^ Such limitations in the films also resulted in a poor fill factor (FF), which can be attributed to increased recombination from an increased series resistance in comparison to the control samples. Further work in optimising smooth deposition are expected to help eliminate the factors limiting performance.

## Experimental

### Precursor synthesis

Unless otherwise stated, the majority of manipulations were performed under nitrogen using standard Schlenk techniques and a Unilab MBraun glove box. Oxygen-free nitrogen (99.9% purity) was obtained from BOC and used as supplied. All solvents were purified by standard methods with respect to oxygen and water and then stored over activated 3 Å molecular sieves until used. Anhydrous nickel(ii) chloride hexahydrate (98+%) was obtained from Acros Organics and used as supplied. Nickel(ii) chloride anhydrous (98%) was obtained from VWR and used as supplied. Dmamp′ (2-dimethylamino-2-methyl-1-propanol) was obtained from MP Biomedicals and purified by standard methods with respect to oxygen and water using the freeze–pump–thaw technique and then stored over 3 Å molecular sieves until used. Methanol (99.8%), ethanol (95%) and petroleum ether (40–60 °C) were purchased from Sigma Aldrich and used as supplied. Tetrahydrofuran (THF) and hexane were obtained from a dry solvent system within the chemistry department at University College London (UCL).

### Precursor analysis

^1^H NMR and ^13^C{^1^H} NMR spectra were recorded using Bruker AMX 300 MHz and Bruker Avance III 600 MHz spectrometers and were referenced to the residual proton and ^13^C resonances of the solvent. Microanalytical data and mass spectrometry data were obtained at UCL. Mass spectra were recorded using Thermo MAT900 and Micromass LCT Premier Spectrometers. Thermogravimetric analysis (TGA) was performed using a Netzsch simultaneous TG-DTA/DSC apparatus equipped with Proteus software at atmospheric pressure, using aluminum pans under a constant flow of helium gas. The heating rate was 10 K min^−1^. X-ray crystallography diffraction data were recorded on an Agilent Super Nova Dual Diffractometer with Cu Kα radiation (*λ* = 1.5418 Å) at 150 K. Using Olex2,^22^ the structure was solved with the SIR2004 (ref. 23) structure solution program using direct methods and refined with the ShelX^24^ refinement package using least squares minimisation. Crystal data for C_6_H_14_NNi_0.5_O (*M* = 145.54 g mol^−1^): orthorhombic, space group *Pbca*, *a* = 7.29116(10) Å, *b* = 10.68510(13) Å, *c* = 17.7933(2) Å, *V* = 1386.22(3) Å^3^, *Z* = 8, *T* = 150.5(7) K, *μ*(CuKα) = 1.965 mm^−1^, *D*_calc_ = 1.395 g cm^−3^, 19 533 reflections measured (15.714° ≤ 2*θ* ≤ 148.974°), 1412 unique (*R*_int_ = 0.0229, *R*_sigma_ = 0.0081) which were used in all calculations. The final *R*_1_ was 0.0217 (*I* > 2*σ*(*I*)) and w*R*_2_ was 0.0610 (all data).

### Synthesis [Ni(dmamp′)_2_]

**Fig. 8:**
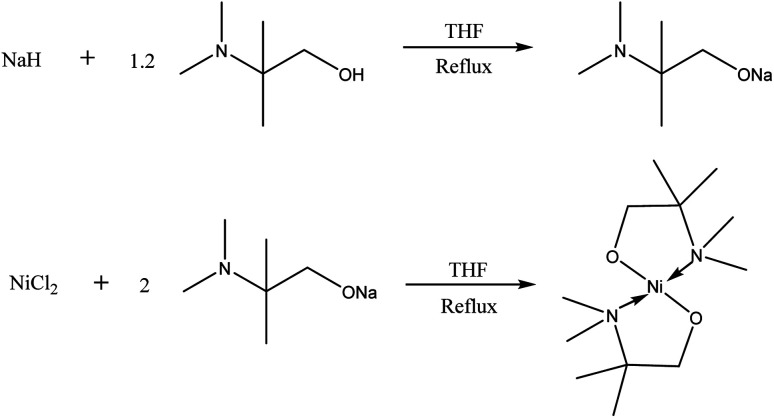
Synthesis of [Ni(dmamp′)_2_].

Sodium hydride (1.745 g, 72.73 mmol) was dissolved in ∼100 mL THF and stirred in an ice bath for 2 hours which formed a milky grey slurry. Dmamp′ (2-dimethylamino-2-methyl-1-propanol) (10.63 mL, 86.17 mmol) was added and the solution was refluxed overnight resulting in a clear orange/brown solution (Fig. 8). The solvent was removed under vacuum to isolate the intermediate salt as a pale yellow solid. The sodium salt was added to a slurry of NiCl_2_ (5.30 g, 40.89 mmol) in ∼50 mL THF at 0 °C and then warmed to room temperature. The solution was then refluxed for 2 days, forming a dark purple/black solution (Fig. 8). The solution was filtered and the volume of solvent reduced by approximately half and left in the freezer overnight. After filtering and removing the solvent under vacuum, a light brown solid was formed. The product was stored under an inert atmosphere. Yield = 10.2 g, 85.9%. Anal. calcd for C_12_H_28_N_2_NiO_2_ (291.06): C, 49.52; H, 9.70; N, 9.62. Found: C, 49.42; H, 9.92; N, 9.36. Mass spectra (CI) *m*/*z*: 291.2 [M]^+^. ^1^H NMR [600 MHz, C_6_D_6_] *δ* 1.19 [singlet, 12H, C–(C*H̲*_3_)_2_], *δ* 2.20 [singlet, 12H, N–(C*H̲*_3_)_2_], *δ* 2.97 [singlet, 4H, O–C*H̲*_2_]. ^13^C{^1^H} NMR [150.90 MHz, C_6_D_6_] *δ* 19.7 [C–(*C̲*H_3_)_2_], *δ* 40.6 [N–(*C̲*H_3_)_2_], *δ* 67.9 [N–*C̲*–(CH_3_)_2_], *δ* 75.8 [O–(*C̲*H_2_)]. Further detail, including spectra, is available in ESI.†

### Thin film synthesis

CVD of nickel oxide thin films were performed using a flow-type, cold-walled reactor described previously.^25^ The reactor was programmed using a custom IGI Systems Lab Interface Input control box which automatically controls all temperatures and heating systems, gas flow rates and solenoid valves. CVD experiments were carried out using [Ni(dmamp′)_2_]. Pureshield argon gas (99.998%) supplied by BOC, was used as the carrier gas for all depositions. Gas flow rates were controlled using Mass Flow Controllers (MFC's) purchased from Brooks Instrument (GF40 model number), with flow rates varying from 20–700 sccm. The reactor running pressures therefore varied in the range of ∼1.0–7.0 mbar. Quartz glass slides (obtained from Wuxi Crystal and Optical Instrument Company Limited) were used as the substrate materials. Prior to deposition, substrates were cut into ∼4.0 × 2.5 cm pieces, cleaned using iso-propanol (Sigma Aldrich, 99.5%) and air dried before loading into the reactor. Whilst the substrate holder was being heated to the required temperature, the reactor was pumped down under vacuum to achieve a base pressure of ∼4 × 10^−2^ mbar. Gas flows were then turned on, where the running pressure was recorded. [Ni(dmamp′)_2_] was introduced into the reaction chamber by passing the carrier gas into the bubbler to assist the transportation of vaporised precursor molecules. To prevent the precursor condensing or reacting in the pipework, the bubbler outlet line was held at a temperature higher than the bubbler temperature but lower than the substrate temperature. Films were deposited by continuously dosing the metal precursor into the reactor under a constant flow of inert gas until the desired reaction time/film thickness had been reached.

### Thin film analysis

Ellipsometry measurements were performed using a Semilab SE-2000 Ellipsometer. A continuous spectrum of light was generated by a broadband 75 W arc lamp including ultraviolet through visible to near infrared (1.25–5 eV). Microspot optics were used confine the beam spot size to 3.5 mm in the minor axis. Data was recorded at angles of incidence of 60, 65, 70 and 75°. Ellipsometric optical models were constructed within the Spectroscopic Ellipsometry Analysis (SEA) software. The NiO model consisted of two components, a Tauc–Lorentz and a Bruggeman dispersion function. An air–NiO diffusion layer (effective medium approximation) was also incorporated in order to account for surface roughness of the films. Fittings were performed within the spectral range 250–990 nm and the obtained refractive indices (*n*) of the deposited films were recorded for comparison at a wavelength of 632.8 nm (1.96 eV). Atomic Force Microscopy (AFM) measurements were obtained using a Nanosurf Easy Scan Atomic Force Microscope, with a 10 μm head in non-contact tapping mode. Scan areas were 5 × 5 μm, with measurements recorded at 250 points per line (20 nm lateral resolution) with 1 s per line scan time. Data extracted from each scan included the arithmetic average roughness (*R*_a_) and the root mean square roughness (*R*_q_ or RMS). X-Ray Diffraction (XRD) measurements were performed using a Bruker-Axs D8 (GaDDS) diffractometer which operates with a Cu X-ray source, monochromated (Kα_1_ and Kα_2_) and a 2D area X-ray detector with a resolution of 0.01°. The diffraction patterns obtained were compared with database standards from the Inorganic Crystal Structure Database (ICSD), Karlsruhe, Germany. The beam spot is approximately 5 mm^2^, which means that several areas of the sample can be analysed separately. For all thin films analysed an incident angle of 0.5–1° was used, and the diffracted X-rays were detected at angles from 10–66°. X-Ray Photoelectron Spectroscopy (XPS) analysis was performed using a Thermo Scientific K-Alpha X-ray photoelectron spectrometer with monochromated Al K alpha radiation, a dual beam charge compensation system and constant pass energy of 50 eV. Survey scans were collected in the range 0–1200 eV. XPS data was fitted using CasaXPS software. The principal peaks of interest were Ti 2p, Ni 2p, O 1s, Si 2p and C 1s. The escape depth in this system was in the range of 1–10 nm. Depth profiling was carried out *via* argon ion sputtering.

### Device fabrication

Fluorine-doped tin oxide coated glass (FTO) was first etched using 2 M hydrochloric acid and zinc followed by sequential cleaning in detergent, deionized water, acetone, ethanol, iso-propanol, and finally treated with oxygen plasma for 10 minutes. A compact layer of NiO was then deposited onto the FTO following a previous report.^20^ Briefly, nickel acetate tetrahydrate (Sigma-Aldrich) was dissolved into 2-methoxyethanol (Sigma-Aldrich, anhydrous), and ethanolamine (Sigma-Aldrich) was used as a stabiliser. The molar ratio between nickel acetate tetrahydrate and ethanolamine was kept at 1 : 1, and the resultant concentration was 0.2 M. The solution was stirred overnight and filtered through a 0.2 μm PTFE filter before deposition. In order to obtain a 20 nm NiO film, the sol–gel was spin coated onto FTO at 4000 rpm and annealed at 250 °C for 20 minutes to achieve a transparent dense film. For the CVD films, NiO films of the same thickness (20 nm) were used as a comparison.

The FTO/NiO substrates were then once again treated with oxygen plasma for 10 minutes before being transferred to a nitrogen filled glovebox. The light absorbing layer, methylammonium lead iodide (MAPbI_3_), was prepared with slight modification to a procedure previously reported by Ahn *et al.*^26^ The CH_3_NH_3_I·PbI_2_·DMSO adduct solution was prepared by mixing 461 mg of PbI_2_ with 159 mg of CH_3_NH_3_I with 600 mg of DMF and 78 mg of DMSO. The solution was stirred for 1 hour at 70 °C and filtered before use. The filtered solution was spin coated on the previously prepared NiO films at 4000 rpm for 30 seconds and after 23 seconds had elapsed, 0.5 mL of diethyl ether was slowly dripped onto the rotating substrate. The transparent CH_3_NH_3_I·PbI_2_·DMSO adduct film was heated to 65 °C for 2 minutes and 100 °C for 10 minutes to obtain a dense CH_3_NH_3_PbI_3_ film. For the electron transport layer, a 23 mg mL^−1^ solution of PCBM (Ossila, 99.5% purity) in chlorobenzene was spin coated on top of the perovskite film at 1500 rpm for 20 seconds. A thin layer of 0.5 mg mL^−1^ BCP (Lumtec, Inc) in methanol was then deposited on top of the PCBM by spin coating at 4000 rpm for 20 seconds. Finally, 100 nm of Ag was thermally evaporated as the counter electrode. *J*–*V* measurements were performed under one sun (AM 1.5G) illumination using a calibrated solar simulator with a xenon lamp (LOT). The light intensity was calibrated by silicon reference cell certificated by National Renewable Energy Laboratory (NREL).

## Conclusions

NiO thin films have been deposited by CVD of the novel nickel dialkylaminoalkoxide precursor [Ni(dmamp)_2_] at substrate temperatures in the range of 250–400 °C, where films with the highest conformality and uniformity tended to be those deposited at 300 °C.

AFM and XRD analysis suggest that the film density and crystallinity increased with film thickness, which was supported by an increase in the refractive indices of the films deposited at 250–350 °C. However at higher growth temperatures (400 °C) films appeared non-uniform and the refractive index decreased, which suggested that the film crystallinity had decreased due to a possible change in film composition and less uniformity in thickness.

Together with the XRD patterns and the AFM images, it can be suggested that films deposited at low growth temperatures/thicknesses consisted of mixtures of amorphous and NiO phases; where growth occurred *via* nucleation sites which grow together and coalesce to form a dense film.

XPS analysis confirmed the presence of Ni^2+^ on the film surfaces with 2p_3/2_ and 2p_1/2_ peak binding energies consistent with those reported in the literature. A prominent satellite shoulder 1.8 eV above the principal 2p_3/2_ peak was observed which is unique to NiO.

In addition to synthesis, we also show that our approach can also be used to integrate NiO in photoelectrodes. While the performance was limited likely due to surface texture (roughness), the un-optimised devices were able to achieve a power conversion efficiency of 3.9%, highlighting their potential application in energy conversion.

## Conflicts of interest

There are no conflicts to declare.

## Supplementary Material

RA-011-D1RA03263A-s001

RA-011-D1RA03263A-s002
