# Supplementary material for: Chemical vapour deposition (CVD) of nickel oxide using the novel nickel dialkylaminoalkoxide precursor [Ni(dmamp′)2] (dmamp′ = 2-dimethylamino-2-methyl-1-propanolate)
Source: RSC Adv. 2021 Jun 23;11(36):22199–205. doi: 10.1039/d1ra03263a (PMC9034214; doi:10.1039/d1ra03263a)
Supplement: RA-011-D1RA03263A-s001 [file RA-011-D1RA03263A-s001.pdf]

Electronic Supplementary Material (ESI) for RSC Advances.
This journal is © The Royal Society of Chemistry 2021

Chemical vapour deposition (CVD) of nickel oxide using the novel nickel dialkylaminoalkoxide precursor [Ni(dmamp')$_2$] (dmamp' = 2-dimethylamino-2-methyl-1-propanolate)

Rachel L. Wilson[1], Thomas J. Macdonald[1,2], Chieh-Ting Lin[3], Shengda Xu[3], Alaric Taylor[4], Caroline E. Knapp[1], Stefan Guldin[4], Martyn A. McLachlan[3], Claire J. Carmalt[1], Chris S. Blackman[1,5]**

Table S1 outlines the crystal data and structure refinement details, and **Table S2** selected bond lengths and bond angles, for [Ni(dmamp')$_2$].

Table S1: Crystal data and structure refinement for [Ni(dmamp')$_2$]

Empirical formula	$C_{12}H_{28}N_2NiO_2$
Formula weight	291.07
Temperature/K	150.5(7)
Crystal system	orthorhombic
Space group	Pbca
a/Å	7.29170(10)
b/Å	10.68490(10)
c/Å	17.7929(2)
α/°	90
β/°	90
γ/°	90
Volume/Å^3	1386.26(3)
Z	4
ρ_{calc}g/cm^3	1.395
μ/mm^{-1}	1.965
F(000)	632.0
Crystal size/mm^3	0.141 × 0.128 × 0.064
Radiation	CuKα (λ = 1.54184)
2Θ range for data collection/°	15.714 to 148.974
Index ranges	$-8 \le h \le 9$, $-13 \le k \le 13$, $-22 \le l \le 22$
Reflections collected	19513
Independent reflections	1410 [R_{int} = 0.0227, R_{sigma} = 0.0078]
Data/restraints/parameters	1410/0/135
Goodness-of-fit on F^2	1.094
Final R indexes [I>=2σ (I)]	R_1 = 0.0209, wR_2 = 0.0566
Final R indexes [all data]	R_1 = 0.0224, wR_2 = 0.0582

Table S2: Selected bond lengths and bond angles for compound (1) [Ni(dmamp')$_2$] (in which ^{1}1-X,1-Y,1-Z).

**Atom - Atom**	**Length (Å)**	**Atom - Atom**	**Length (Å)**
**Ni(1) – O(1)**	1.8427(7)	**N(1) – C(1)**	1.5239(12)
**Ni(1) – O(1)^1**	1.8427(7)	**N(1) – C(5)**	1.4788(13)
**Ni(1) – N(1)^1**	1.9545(8)	**C(3) – C(1)**	1.5319(14)
**Ni(1) – N(1)**	1.9546(8)	**C(4) – C(1)**	1.5311(14)
**O(1) – C(2)**	1.3992(12)	**C(1) – C(2)**	1.5313(14)
**N(1) – C(6)**	1.4896(13)		
**Atom – Atom - Atom**	**Angle (°)**	**Atom – Atom - Atom**	**Angle (°)**
**O(1) – Ni(1) – O(1)^1**	180.00	**C(5) – N(1) – Ni(1)**	113.89(7)
**O(1)^1 – Ni(1) – N(1)^1**	88.43(3)	**C(5) – N(1) – C(6)**	106.95(8)
**O(1) – Ni(1) – N(1)**	88.43(3)	**C(5) – N(1) – C(1)**	112.91(8)
**O(1)^1 – Ni(1) – N(1)**	91.57(3)	**N(1) – C(1) – C(3)**	108.98(8)
**O(1) – Ni(1) – N(1)^1**	91.57(3)	**N(1) – C(1) – C(4)**	113.07(8)
**N(1) – Ni(1) – N(1)^1**	180.0(5)	**N(1) – C(1) – C(2)**	104.13(8)
**C(2) – O(1) – Ni(1)**	112.10(6)	**C(3) – C(1) – C(4)**	110.37(9)
**C(6) – N(1) – Ni(1)**	105.98(6)	**C(2) – C(1) – C(3)**	108.84(8)
**C(6) – N(1) – C(1)**	112.61(8)	**C(2) – C(1) – C(4)**	111.22(8)
**C(1) – N(1) – Ni(1)**	104.41(6)	**O(1) – C(2) – C(1)**	110.15(8)

Film thickness was obtained from the ellipsometry measurements and showed an approximately linear relationship of increasing film thickness with deposition temperature (**Table S3**).

Table S3: Film thickness and refractive index values of NiO films deposited by CVD of [Ni(dmamp')$_2$] at various temperatures

**Deposition time (hours)**	**Growth temp (°C)**	**Film thickness (nm)**	***n* @ 632.8 nm**
**24**	250	17	1.5

**24**	300	40	2.3
**24**	350	85	2.6
**24**	400	117	1.8

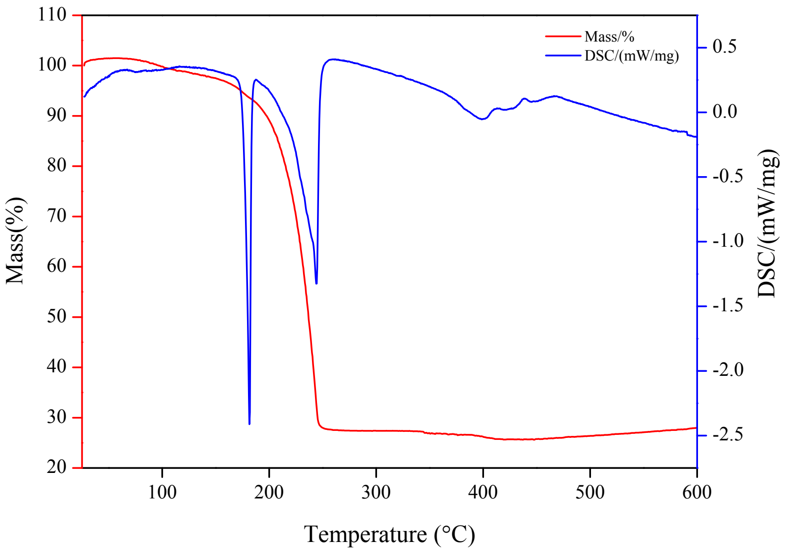

Figure S1. TGA/DSC curves for [Ni(dmamp')$_2$].

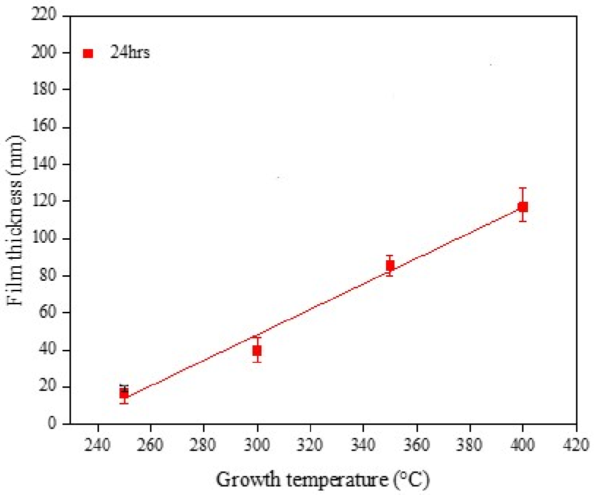

Figure S2: Temperature dependence on the film thickness of NiO films deposited by CVD of [Ni(dmamp')$_2$] grown for a given time of 24 hours.

Films deposited at a growth temperature of 400 °C appeared visually non-uniform in thickness, with the inlet end of the substrate thicker than the outlet end of the substrate. Reduction of the growth temperature from 400 °C to 300 °C resulted in visually more uniform thin films with refractive index values more consistent with literature values for NiO. As a growth temperature of 300 °C appeared to provide visually uniform films with low roughness, subsequently the relationship between deposition time and NiO film thickness was investigated at this temperature (**Figure S3**).

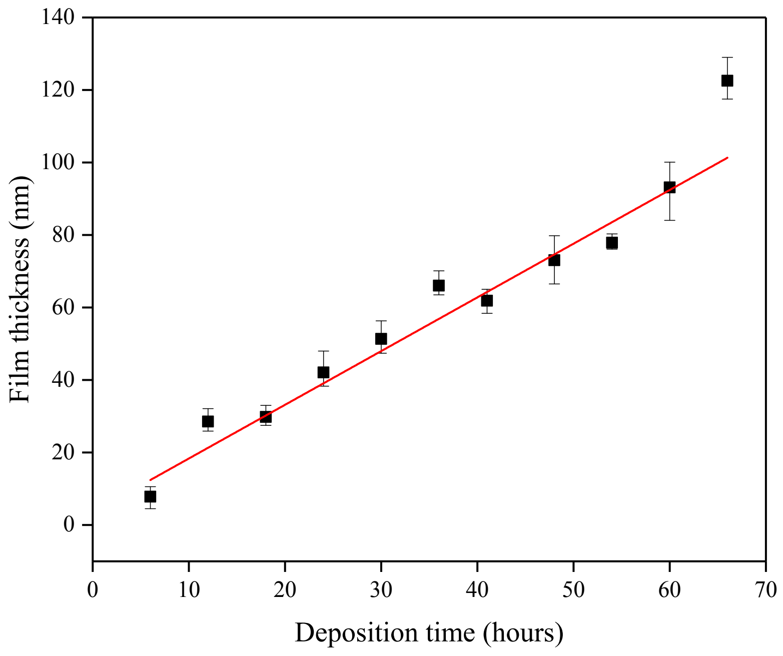

Figure S3: Relationship between deposition time and film thickness for NiO films deposited by CVD of [Ni(dmamp')$_2$] at a single growth temperature of 300 °C.

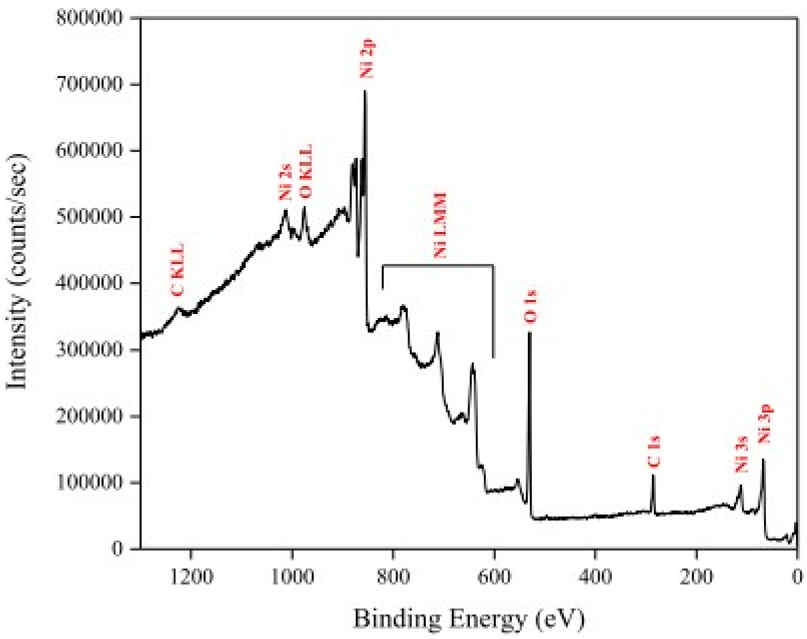

Figure S4: Typical XPS survey spectrum of NiO films deposited by CVD of [Ni(dmamp')$_2$] at a growth temperature of 300 °C.

High resolution surface scans (Figure S5a) of the Ni2p peak confirm the presence of Ni^{2+}, with $2p_{3/2}$ and $2p_{1/2}$ peak binding energies of 855.4 eV and 873.2 eV respectively, with a peak separation of 17.8 eV. These values are within ± 0.2 eV of literature values.[1] Characteristic satellite peaks for the $2p_{3/2}$ and $2p_{1/2}$ peaks were observed at 861.8 ± 0.2 eV and 880.2 ± 0.2 eV respectively. The prominent satellite shoulder 1.8 eV above the Ni $2p_{3/2}$ principal peak is unique to NiO.[2]

De-convolution of the O1s peak reveals 2 peaks. The two peaks at higher binding energy, 532.8 ± 0.2 eV and 531.4 ± 0.2 eV can be attributed to surface bound carbon and surface adsorbed water, respectively. The peak with the lowest binding energy (529.6 ± 0.2 eV) is attributed to the O1s core peak of O^{2-} bound to Ni^{2+} (Figure S5b). Again, these peaks are consistent with literature values for these peak environments.

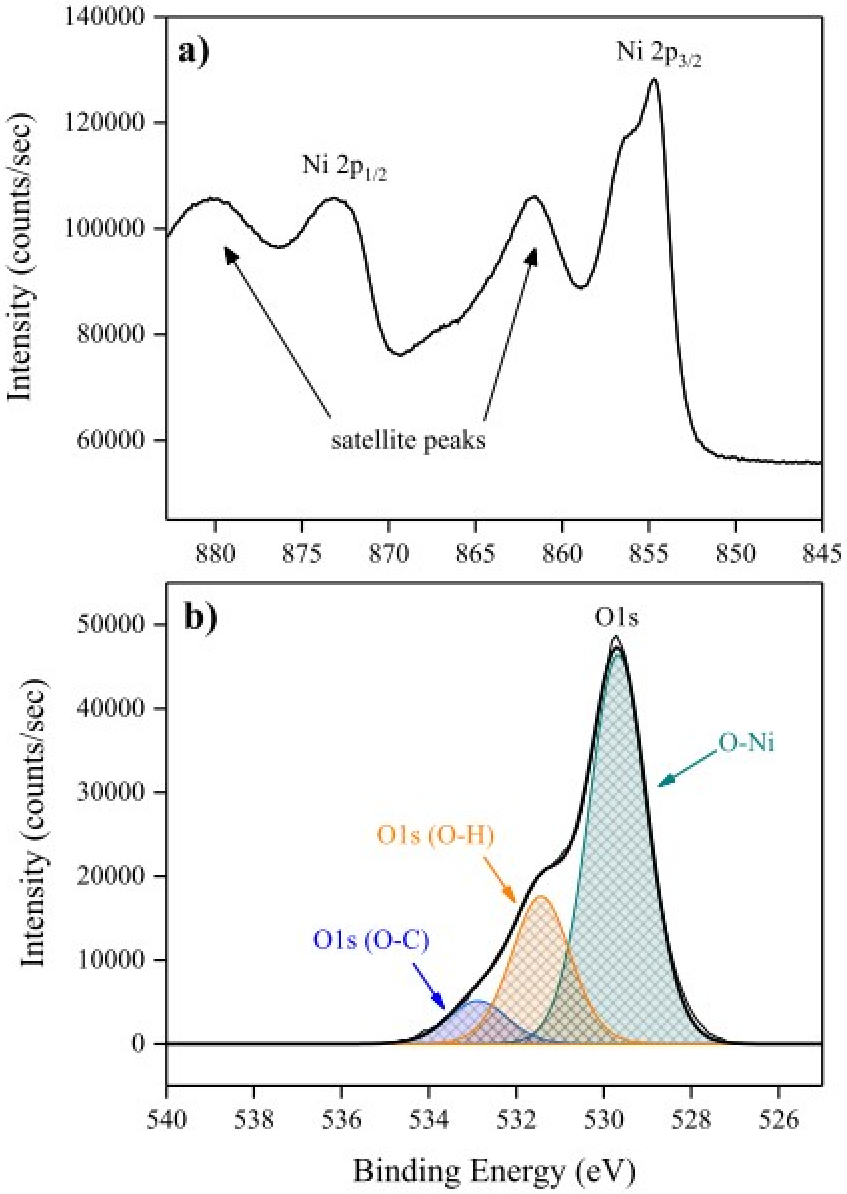

Figure S5: Typical high resolution surface XPS spectra of a) Ni2p peak and b) de-convoluted O1s peak for NiO films deposited by CVD of [Ni(dmamp')$_2$] at a growth temperature of 300 °C.

Table S4: photovoltaic parameters for NiO control and NiO CVD devices.

**Photoelectrode**	J_{SC} (mA cm^{-2})	V_{OC} (V)	***FF***	**PCE** (%)
NiO Control	21.3 (+/- 0.1)	1.04 (+/- 0.001)	0.66 (+/- 0.05)	14.1 (+/- 0.1)
NiO CVD	7.8 (+/- 0.5)	1.02 (+/- 0.01)	0.50 (+/- 0.5)	3.9 (+/- 0.2)

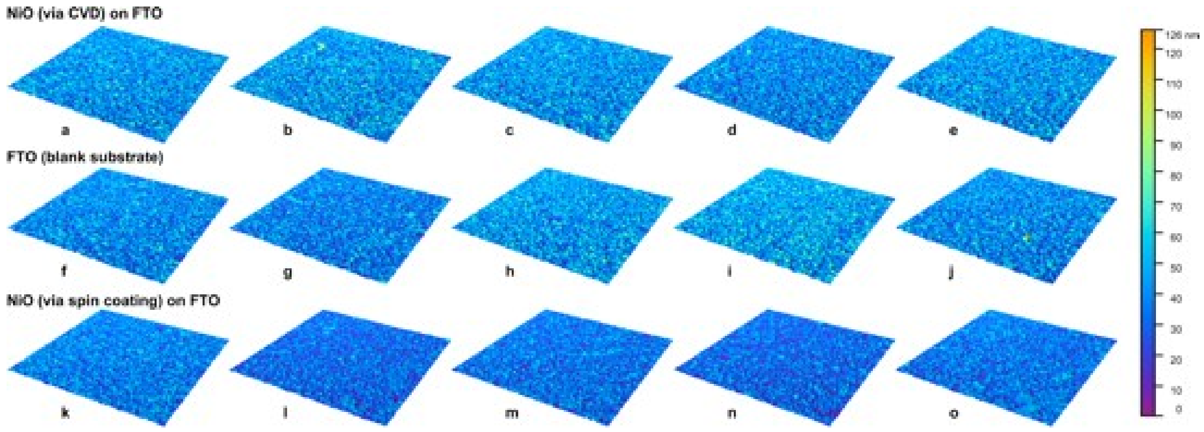

Figure S6: AFM images of 5x5 µm areas over a lateral resolution of 10 nm for five duplicate samples of FTO, CVD-deposited NiO/FTO and spin-coating-deposited NiO/FTO.

Table S5: AFM data of 5x5 µm areas over a lateral resolution of 10 nm for five duplicate samples of FTO, CVD-deposited NiO/FTO and spin-coating-deposited NiO/FTO.

**Aggregated (Mean)**			
**Sample**	**Z range (nm)**	**Average value (nm)**	**RMS roughness (Sq, nm)**
**FTO blank substrate**	97	42	11
**NiO (via CVD) on FTO**	100	43	12
**NiO (via spin.) on FTO**	73	35	9.7
**Aggregated (Standard Deviation)**			
**Sample**	**Z range (nm)**	**Average value (nm)**	**RMS roughness (Sq, nm)**
**FTO blank substrate**	17	3.4	0.24
**NiO (via CVD) on FTO**	10	1.6	0.47
**NiO (via spin.) on FTO**	2.9	2.5	0.13

^{1}H NMR and ^{13}C{^{1}H} NMR spectra for [Ni(dmamp')$_2$] are shown in Figure S7 and Figure S8 respectively. The ^{1}H NMR spectrum shows 3 singlet proton environments at 1.19 ppm, 2.20 ppm and 2.97 ppm, corresponding to 4 methyl groups on the ligand backbone (Ha), 4 methyl groups on the nitrogen atoms (Hb) and 2 ethyl groups on the ligand backbone (Hc), respectively. The carbon NMR spectrum again shows 4 different peak environments at 19.7, 40.6, 67.9 and 75.8 ppm, which are associated to the carbon environments C1, C2, C3 and C4, respectively as shown in Figure S8.

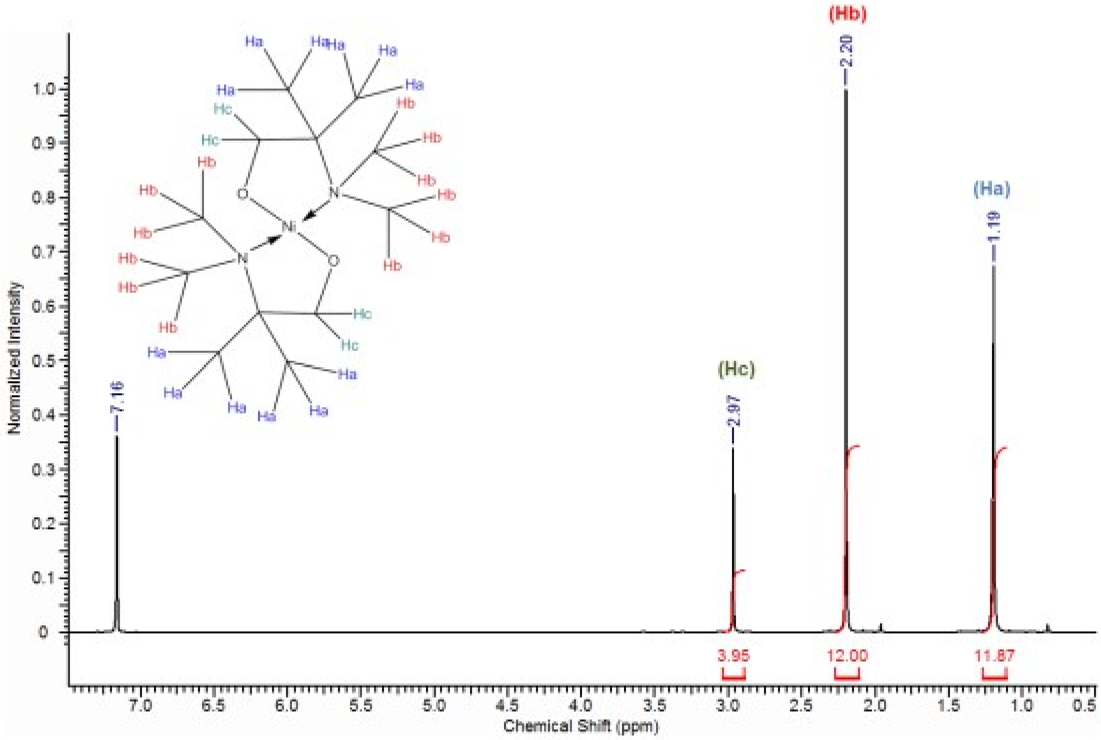

Figure S7: ^{1}H NMR spectrum of $[Ni(dmamp')_2]$

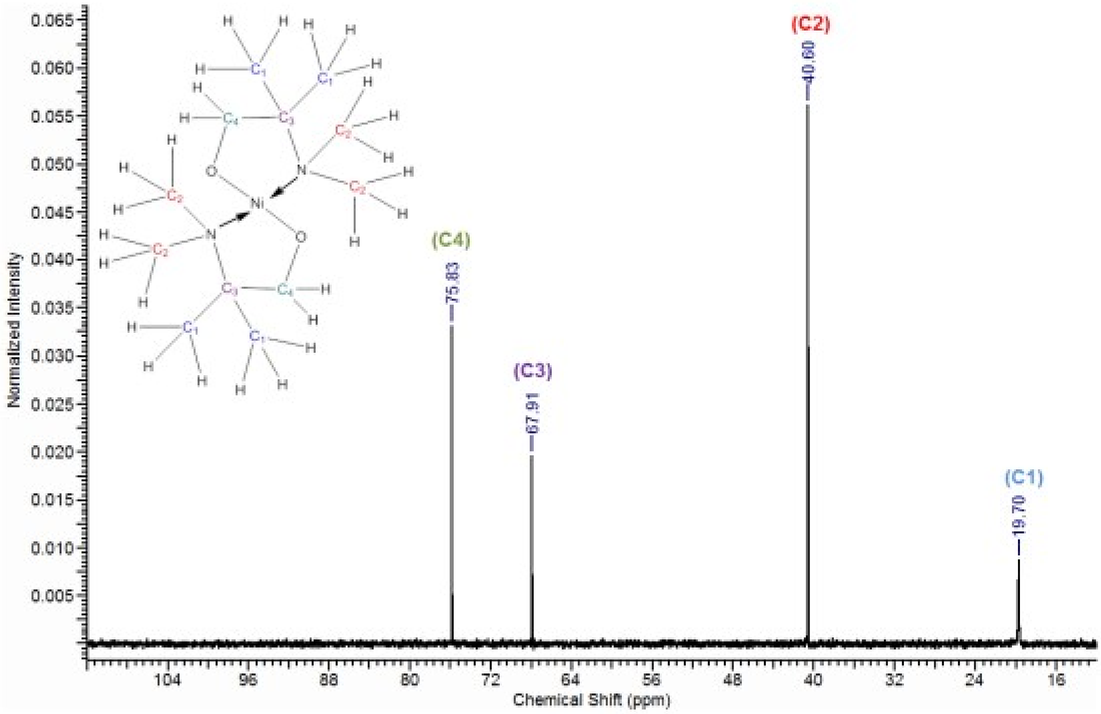

Figure S8: ^{13}C NMR spectrum of $[Ni(dmamp')_2]$

References:

1. K.-C. Min, M. Kim, Y.-H. You, S. S. Lee, Y. K. Lee, T.-M. Chung, C. G. Kim, J.-H. Hwang, K.-S. An, N.-S. Lee, Y. Kim, *Surf. Coatings Technol.*, 2007, **201(22-23)**, 9252-9255.

2. N. S. McIntyre, M. G. Cook, *Anal. Chem.*, 1975, **47(13)**, 2208-2213.
